# Two Novel Myelin Protein Zero Mutations in a Group of Chinese Patients

**DOI:** 10.3389/fneur.2021.734515

**Published:** 2021-12-02

**Authors:** Bin Chen, Zaiqiang Zhang, Na Chen, Wei Li, Hua Pan, Xingao Wang, Yuting Ren, Yuzhi Shi, Hongfei Tai, Songtao Niu

**Affiliations:** ^1^Department of Neurology, Beijing Tiantan Hospital, Capital Medical University, Beijing, China; ^2^China National Clinical Research Center for Neurological Diseases, Beijing, China; ^3^Monogenic Disease Diagnosis Center for Neurological Disorders, Precision Medicine Research Center for Neurological Disorders, Beijing, China

**Keywords:** myelin protein zero, Charcot-Marie-Tooth disease, spectrum, Chinese, Japanese, Koreans

## Abstract

Mutations in the myelin protein zero gene are responsible for the autosomal dominant Charcot-Marie-Tooth disease (CMT). We summarized the genetic and clinical features of six unrelated Chinese families and the genetic spectrum of Chinese patients with myelin protein zero (*MPZ*) mutations. Our study reports data from a group of Chinese patients consisting of five males and one female with the age of disease onset ranging from 16 to 55 years. The initial symptom in all the patients was the weakness of the lower limbs. Electrophysiological presentations suggested chronic progressive sensorimotor demyelinating polyneuropathy. Overall six mutations were identified in the cohort, including four known mutations [c.103G>T (p.D35Y), c.233C>T (p.S78L), c.293G>A (p.R98H), and c.449-1G>T], and two novel mutations [c.67+4A>G with a mild CMT1B phenotype, and (c.79delG) p.A27fs with a rapidly progressive CMT1B phenotype]. According to the literature review, there are 35 Chinese families with 28 different *MPZ* mutations. The *MPZ* mutational spectrum in Chinese patients is very heterogeneous and differs from that of Japanese and Korean individuals, although they do share several common hot spot mutations.

## Introduction

Charcot-Marie-Tooth disease is the most common inherited disorder of the peripheral nervous system (PNS). It is characterized by slow-progressing weakness, muscle atrophy, and sensory impairment, with the symptoms being most evident in the distal part of the legs (1). Charcot-Marie-Tooth disease (CMT) is primarily classified into two types based on electrophysiological findings. A median motor nerve conduction velocity (NCV) of ≤ 38 m/s indicates CMT1 while NCV of >38 m/s with a reduced compound muscle action potential (cMAP) indicates CMT2 (1, 2). CMTs is genetically determined disorders involving nearly 100 genes (3).

Myelin protein zero (P0, *MPZ*) is the most abundant protein in peripheral myelin and is produced by Schwann cells (4). It is a member of the immunoglobulin supergene family and functions as an adhesion molecule that mediates the compaction of the PNS (5). *MPZ* mutations are responsible for autosomal dominant CMT, which can be divided into CMT1B, CMT2I, and Dejerine-Sottas syndrome, based on the clinical and electrophysiological characteristics. These are found in 4.1–5% of all CMT patients (6).

Almost 300 mutations in *MPZ* have been identified (7). The early onset (infantile and childhood) phenotypes likely represent developmentally impaired myelination, whereas the adult-onset phenotypes reflect axonal degeneration without antecedent demyelination (8). Previous studies have shown that the spectra and frequencies of *MPZ* mutations in Caucasian and Japanese cohorts are different (7). Several studies have reported Chinese patients with *MPZ* mutations, but it is currently unknown whether there are differences in the spectra of *MPZ* mutations between Chinese and other ethnicities (9–20). Herein, we report the mutational spectrum and clinical features of six unrelated Chinese families with *MPZ* in our hospital over a 7 years period.

## Methods

### Patients

We enrolled six probands from 76 unrelated Chinese families who visited the Beijing Tiantan Hospital from January 2012 to August 2019 with suspected CMT or related mutations in CMT genes as detected by targeted next-generation sequencing (NGS). All participants provided informed consent for this study, and ethical approval was obtained from the Human Research Ethics Committee of Beijing Tiantan Hospital. All patients were examined and evaluated by the participating neurologists. Their phenotypes were retrospectively defined based on clinical manifestations, family histories, and electrophysiological data, collected from the patient medical records. Nerve conduction studies were performed using standard techniques and a Medelec MS25 electromyograph (Mistro, Surrey, United Kingdom).

### Molecular Analysis

Genomic DNA was extracted from peripheral venous blood samples from the six probands and available family members following standard procedures. All patients were negative for 17p12 (*PMP22*) duplication and deletion. The NGS panel covered all the exons and flanking sequences of genes that were known to be associated with hereditary neuropathies (21). Sanger sequencing of the variants and co-segregation analysis were conducted for all patients and available family members.

The identified variants were determined using the databases of genomic variants, including the 1,000 Genomes Project, the Genome Aggregation Database (gnomAD), and The Single Nucleotide Polymorphism Database (dbSNP). A database of 8,000 healthy controls of Chinese origin was also screened. The biological relevance of the novel amino acid changes was studied using the Mutation Taster (https://www.mutationtaster.org) and SIFT-Indels (https://sift.bii.a-star.edu.sg/www/SIFT_indels2.html). Splice site mutations were predicted using Human Splicing Finder (HSF) software 3 (http://www.umd.be/HSF3/HSF.shtml) and single nucleotide variants within splicing consensus regions (scSNV).

## Results

### Clinical Data

The probands were from six unrelated families and included five males and one female. The age of disease onset for five patients was >40 years. The first symptom in all patients was distal weakness in the lower limbs. Three patients (1, 5, and 6) had a family history of peripheral neuropathy, which is characterized by an autosomal dominant inheritance. At the age of 43 years, patient 1 started showing weakness in both the lower limbs. Three years after the first symptoms appeared, the patient had slower walking than before. The father of the patient also showed lower limb weakness in his 40s. Patient 5 had a history of abnormal gait for about 3 years. His mother and sister showed similar symptoms. Patient 6 suffered from ankle sprains at the age of 16 years, and their grandfather and father also developed an abnormal gait in their 40s. Family members of the remaining patients were not available for testing. Patient 2 had weakness in his lower limbs for approximately 14 years, with a rapid progression of the ailment in the past 2 years. Patient 3 had a history of weakness in both the lower limbs for approximately 11 years and started showing weakness in the hands for the past 2 years. Patient 4 has had a history of abnormal gait for the past 12 years. All probands displayed distal limb muscle atrophy with pes cavus.

Blood creatine kinase levels were normal in all the patients except patients 2 and 3 [patient 2 (360 U/L) and patient 3 (566 U/L); normal range 24–194 U/L] However, patient 2 had normal levels of cerebrospinal fluid protein [31 mg/dl (normal range 15–45 mg/dl)] and cell count 3/μl (normal range 0–5). The remaining five patients did not undergo lumbar puncture. The clinical data of six CMT patients with *MPZ* mutations are summarized in Table 1.

**Table 1:** Clinical features of six CMT patients with *MPZ* mutations.

**Proband**	**Gender/age**	**Age of onset**	**Muscle weakness (UL/LL)**	**Paresthesia (UL/LL)**	**Nucleotide change**	**Amino acid change**
1	M/50	43	+/++	–/+	c.67+4A>G	5-splice site
2	M/64	50	+/+++	–/+	c.79delG	p. A27fs
3	M/63	52	++/++	–/+	c.293 G>A	p. R98H
4	M/67	55	+/++	–/+	c.103 G>T	p. D35Y
5	M/49	46	+/++	–/+	c.233 C>T	p. S78L
6	F/19	16	+/+	–/–	c.449-1 G>T	3-splice site

The electrophysiological results from six patients demonstrated a sensorimotor demyelinating polyneuropathy with multiple motor nerves showing prolonged distal latencies. The F-wave persistence rate of the bilateral tibial nerves was 65%, with significantly prolonged latencies in patient 2. The nerve conduction studies of six patients are summarized in Table 2. Based on their clinical and electrophysiological features, all patients were diagnosed with CMT1 according to the diagnostic criteria of CMT.

**Table 2:** Nerve conduction velocity results of six patients.

		**Motor nerve conduction** **CMAP (mV)/MNCV (m/s)**				**Sensory nerve conduction** **SNAP (μV)/SNCV (m/s)**			
		**Median**	**Ulnar**	**Tibial**	**Peroneal**	**Median**	**Ulnar**	**Tibal**	**Sural**
Patient 1	R	3.4/23.6	2.1/28.8	NE	0.8/17.0	3.2/26.8	NE	NE	ND
	L	3.8/24.7	2.5/29.7	NE	0.1/21.2	3.9/27.6	NE	NE	ND
Patient 2	R	3.6/37.4	7.1/38.6	0.5/33.4	7.0/29.9	10.3/32.0	4.8/33.1	NE	NE
	L	3.4/37.2	6.9/33.7	0.5/29.6	5.2/30.1	11.1/33.1	18/35.8	NE	NE
Patient 3	R	9.9/18.3	4.1/18.2	1.4/14.2	NE	3.8/19.8	NE	NE	NE
	L	11.0/26.2	15.7/17.2	ND	ND	ND	ND	NE	NE
Patient 4	R	5.9/34.1	4.3/36.2	4.4/24.9	3.9/26.4	3.4/28.6	4.6/31.7	NE	NE
	L	4.8/28.7	3.1/29.9	4.1/28.5	3.4/31.4	3.9/26.3	ND	ND	ND
Patient 5	R	3.6/34.4	2.9/37.6	1.8/30.8	1.3/25.2	4.3/39.8	4.0/41.3	NE	NE
	L	ND	ND	ND	ND	ND	ND	NE	ND
Patient 6	R	ND	ND	8.8/36.6	ND	43.0/43.3	13.6/46.1	1.2/27.8	1.8/25.0
	L	8.0/33.3	10.0/42.6	7.8/36.2	4.6/33.1	58.9/45.2	11.0/45.3	NE	NE

*CMAP, compound muscle action potential; MNCV, motor nerve conduction velocity; SNAP, sensory nerve action potential; SNCV, sensory nerve conduction velocity; NE, not elicited; ND, not done; R, right side; L, left side*.

### Genetic Data

**Figure 1:**
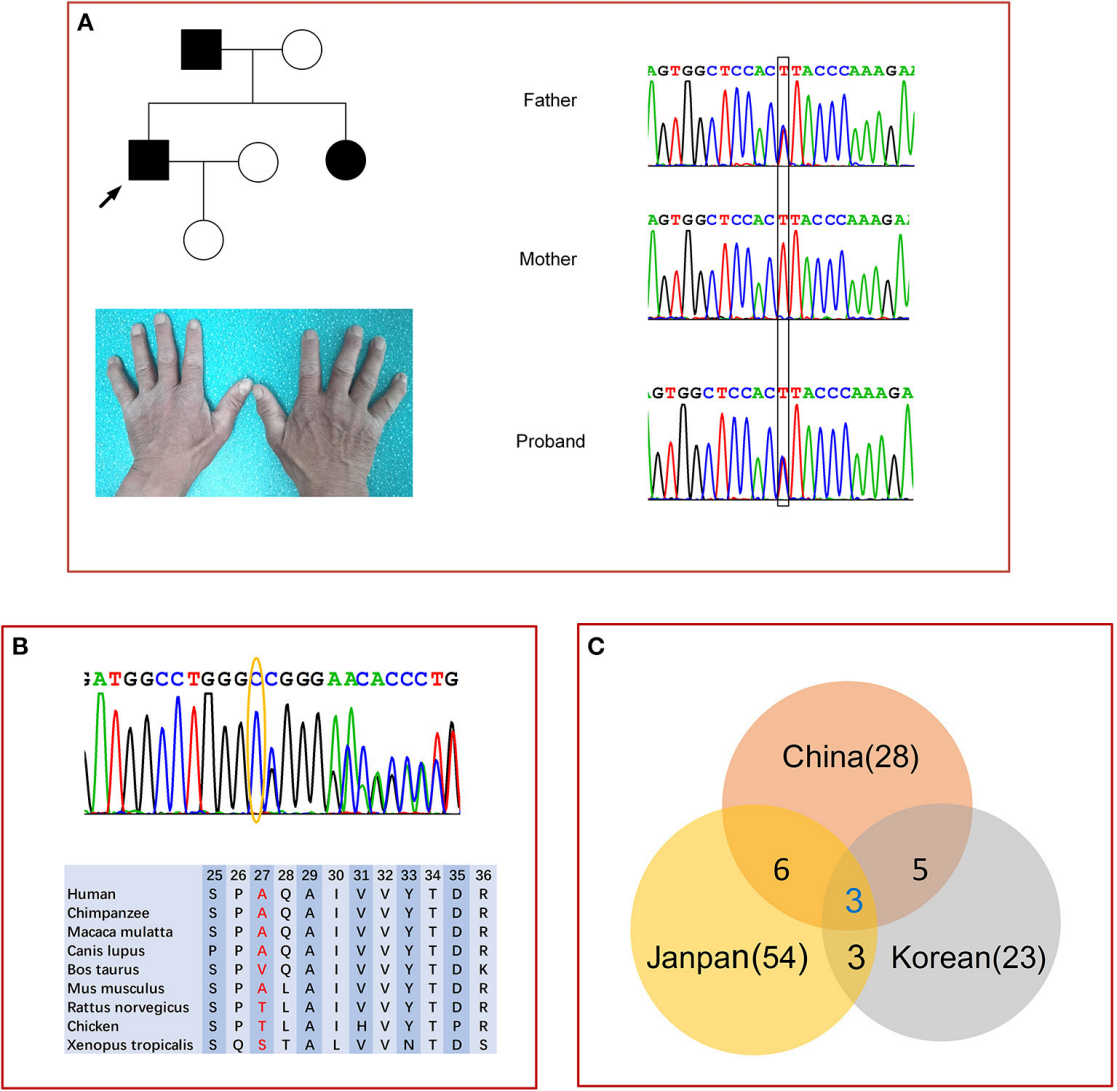
Genetic analysis of *MPZ*. **(A)** Pedigree and sequencing chromatograms of family 1 with c.67+4A>G variants. Circle, female; square, male; filled symbol, patient. The bases in the square frame are mutational sites. Mild muscle atrophy in the first dorsal interosseous muscle of patient 1. **(B)** Sanger sequencing results confirm the guanine deletion mutation at nucleotide position 79 (yellow oval) in *MPZ* exon 2 of patient 2, and the adjacent amino acid residues among different species. **(C)** Venn diagram of the *MPZ* mutational spectra in three countries.

We identified six *MPZ* mutations in six probands which were verified by Sanger sequencing, these included four known mutations [c.103G>T (p.D35Y), c.233C>T (p.S78L), c.293G>A (p.R98H), and c.449-1G>T], (7, 8) a novel frameshift variant c.79delG (p. A27fs) in patient 2, and a novel intronic splice site variant (c.67+4A>G) in patient 1 and his father (Figure 1A). Both variants were absent from the controls (1,000 Genomes, gnomAD, dbSNP, and 8,000 healthy Chinese controls). The c.79delG in exon 2 leads to a shift in the open reading frame and an amino acid change at position 46 from valine (GTG) to a stop codon (TGA), which causes translation termination. p.A27 and adjacent amino acid residues are highly conserved among different animal species (Figure 1B). Mutation Taster and SIFT-Indels predicted that the c.79delG is probably a disease-causing mutation. According to the ACMG Standards, c.79delG is considered a pathogenic variant due to the evidence of pathogenicity for PVS1 (loss of protein function), PM2 (absent from controls), PP3 (harmful effects on gene or gene products). The c.67+4A>G variant is located within the highly conserved intron 1 and may most likely affect splicing by HSF and cause abnormal splicing by abolishing the donor splice site of exon 2 (Ada-score = 0.996, RF-score = 0.846) by scSNV. The c.67+4A>G variant was predicted to be a likely pathogenic variant due to PM1 (located in p0 C-terminal domain), PM2 (absent from controls), PP1 (coseparation in the family), PP3 (harmful effects on gene or gene products), according to ACMG.

## Discussion

In this study, we investigated six probands with inherited peripheral neuropathies associated with *MPZ* variants, who visited a single medical center. We detected two novel variants that are likely to induce a pathogenic phenotype. The c.67+4A>G variant was confirmed in an affected proband and cosegregation with similar peripheral neuropathy in the affected father. The p.A27fs mutation resulted in premature truncation of the protein, which led to a significant change in the protein structure and function. Both novel variants were not seen in healthy individuals with no known history of neurogenetic diseases or in multiple databases, which further suggested that c.67+4A>G and p.A27fs are disease-causing rather than normal variants of *MPZ*.

Our study further confirmed that *MPZ* mutations are associated with specific phenotypes, especially at the age of onset (8). In general, CMT1B patients in this study with *MPZ* mutations had relatively mild clinical phenotypes. The patient with the p.A27fs mutation showed a relatively rapid progression in the late stage of the disease, although a report showed frameshift mutation in *MPZ* caused a mild clinical phenotype (22). We found that the age of onset of patients with S78L and R98H mutations was during adulthood rather than during childhood or infancy (8). The clinical manifestations of *MPZ* mutations varied among the enrolled families. We also found mild elevations in serum CK levels in some patients and a normal CSF protein level in the CMT1B patient (patient 2) (7).

We searched the PubMed database using the terms “*MPZ”* and “Chinese” and excluded patients who may have been associated with previous reports based on the research team and publication time. We then summarized the *MPZ* mutational spectra and clinical characteristics of 35 families of unrelated Chinese origin (9–20). The total mutations summarized in the Chinese patients with *MPZ* mutations are shown in Table 3. According to the literature review, there were 35 known cases of unrelated Chinese families from mainland China and Taiwan presenting these mutations. To date, 28 different *MPZ* mutations have been identified in Chinese individuals. Overall, mutations in *MPZ* are heterogeneous in Chinese individuals without founder mutations. The most common type of *MPZ* mutation in Chinese individuals is the missense mutation (21/28,75%) followed by frameshift mutation (5/28,17.8%), and lastly, splice site mutation in introns (2/28,7.2%). The findings showed that in Chinese patients, 46% of the mutations occurred in exon 3, and 31% in exon 2. The R98H and R98C mutations have been found in 14% (5/35) of Chinese families, from North China and South China, along with those from Taiwan (9, 10, 16).

**Table 3:** *MPZ* mutations in the 35 Chinese families.

**Exon**	**Amino acid change**	**Families**	**Age of onset**	**References**
2	P26L	1	Neonate	(9)
2	A27fs	1	Adult	This report
2	I30M	2	<2 NA	(10) (11)
2	T34N	1	NA	(11)
2	D35Y	1	Adult	This report
2	V58D	1	Childhood	(12)
2	S63F	1	Childhood/Adult	(12)
2	T65I	2	Childhood	(12, 13)
2	S78L	1	Adult	This report
3	H81L	1	Adult	(13)
3	R98H	3	Adult NA	(12), This report (11)
3	R98C	2	Childhood	(10), (12)
3	D121N	1	Adult	(14)
3	G123S	1	Adult	(15)
3	T124M	1	Adult	(13)
3	D128G	1	NA	(16)
3	K130R	2	Neonate NA	(9) (11)
3	P132A	1	Childhood	(10)
3	I135M	1	Adult	(17)
3	S140C	1	Adult	(13)
3	F147S	1	Childhood	(13)
4	Q187fs	1	Adult	(17)
5	K211SfsX41	1	Neonate	(9)
6	H225Qfs*10	1	Adult	(18)
6	S233fs	1	NA	(16)
6	D246G	1	Childhood	(19)
Intron	c.449-1G>T	2	Childhood NA	This report (20)
Intron	c.67+4A>G	1	Adult	This report

*NA, not available*.

Fifty-four different mutations in the *MPZ* have been reported in Japanese individuals (1, 7, 23–26) and 23 mutations in Korean individuals (27, 28). Among them, only six identical mutations (D35Y, S78L, R98C, R98H, T124M, K130R) were found in both Chinese and Japanese patients, (7) and five identical mutations (S78L, R98C, T124M, P132A, c.449- 1G>T) were found in both Chinese and Korean families (Figure 1C) (27, 28). T124M in *MPZ* could be found in 8–17% of Japanese CMT families, but only in 3% of Chinese families. Interestingly, the R98H mutation has the highest *MPZ* mutation frequency in Japan (7). Mutations that were not detected in Japan could also not be found in China. The c.449-1G>T mutation was identified in 13.8% (5/36) of the affected Korean families and 5.7% (2/35) of Chinese families (27, 28).

In conclusion, we identified six *MPZ* mutations including two novel mutations in this study. Patients with c.67+4A>G mutations tended to have relatively milder clinical manifestations. Patients with p.A27fs mutation tended to have a rapid progression in the late stage of the disease. *MPZ* mutations in Chinese individuals were very heterogeneous. The frequency and spectrum of *MPZ* mutations in Chinese individuals are different from those in Japanese and Koreans, although common hot spot mutations exist among these ethnic groups. This report will help with genetic and clinical studies of Chinese CMT patients with *MPZ* mutations.

## Data Availability Statement

The datasets presented in this article are not readily available due to ethical or privacy restrictions. Requests to access the datasets should be directed to the corresponding author.

## Ethics Statement

The studies involving human participants were reviewed and approved by Human Research Ethics Committee of Beijing Tiantan Hospital. The patients/participants provided their written informed consent to participate in this study. Written informed consent was obtained from the individual(s) for the publication of any potentially identifiable images or data included in this article.

## Author Contributions

BC, SN, and ZZ designed the study and wrote the paper. SN, XW, HT, YR, YS, and BC analyzed the clinical data. NC and HP analyzed the electrophysiological data. BC and WL analyzed the genetic results. All authors have read and approved the manuscript.

## Conflict of Interest

The authors declare that the research was conducted in the absence of any commercial or financial relationships that could be construed as a potential conflict of interest.

## Publisher's Note

All claims expressed in this article are solely those of the authors and do not necessarily represent those of their affiliated organizations, or those of the publisher, the editors and the reviewers. Any product that may be evaluated in this article, or claim that may be made by its manufacturer, is not guaranteed or endorsed by the publisher.
